# *OsDPE2* Regulates Rice Panicle Morphogenesis by Modulating the Content of Starch

**DOI:** 10.1186/s12284-023-00618-3

**Published:** 2023-02-03

**Authors:** Yi Zheng, Debao Fu, Zenan Yang

**Affiliations:** 1 National Key Laboratory of Crop Genetic Improvement, Huazhong Agricultural University, Wuhan, China; 2 Hubei Hongshan Laboratory, Wuhan, China

**Keywords:** DPE2, Starch, Rice, Panicle morphogenesis, *LAX1*

## Abstract

Starch is a carbon sink for most plants, and its biological role changes with response to the environment and during plant development. Disproportionating Enzyme 2 (DPE2) is a 4-α-glycosyltransferase involved in starch degradation in plants at night. *LAX1* plays a vital role in axillary meristem initiation in rice. Herein, results showed that *Oryza sativa Disproportionating Enzyme 2* (*OsDPE*2) could rescue the mutant phenotype of *lax1-6*, *LAX1* mutant. *OsDPE2* encodes rice DPE2 located in the cytoplasm. In this study, OsDPE2 affected the vegetative plant development of rice via DPE2 enzyme. Additionally, OsDPE2 regulated the reproductive plant development of rice by modulating starch content in young panicles. Furthermore, haplotype OsDPE2(AQ) with higher DPE2 enzyme activity increased the panicle yield of rice. In summary, OsDPE2 can regulate vegetative and reproductive plant development of rice by modulating starch content. Furthermore, DPE2 activities of OsDPE2 haplotypes are associated with the panicle yield of rice. This study provides guidance for rice breeding to improve panicle yield traits.

**Supplementary Information:**

The online version contains supplementary material available at 10.1186/s12284-023-00618-3.

## Introduction

Starch is a carbon sink for most plants, and its biological role changes with response to the environment and during plant development (Eberhard et al. 2008; Gibon et al. 2004; Lloyd and Kossmann 2015; Lu et al. 2005; Scofield et al. 2009; Sulpice et al. 2014). Disproportionating Enzyme 2 (DPE2), a 4-α-glycosyltransferase, can cleave the α-1, 4-glucoside bond to release α-1, 4-glucoside, which is then transferred to the non-reducing end of an existing α-1, 4-linked chain to form a new α-1, 4-glucoside bond (Chia et al. 2004). Non-functional DPE2 causes excessive maltose accumulation in the leaves, thus inhibiting plant growth.

Rice (*Oryza sativa*) is an important cereal crop, feeding almost half of the global population. Studies on the regulatory network of rice yield traits have markedly improved rice breeding (Huang et al. 2016). The morphological structure of the rice panicle exhibits polymorphic meristem growth and development (Bommert et al. 2005; Furutani et al. 2006). *LAX1*, encoding a basic helix-loop-helix transcription factor, is expressed in the boundary between the shoot apical meristem and the region of new meristem formation (Komatsu et al. 2003; Oikawa and Kyozuka 2009; Tabuchi et al. 2011). Haplotype analysis has revealed *LAX1* gene sequence differences between *indica* and *japonica* (Abbai et al. 2019). Moreover, a recent correlation analysis of the key locus responsible for the variation and yield traits of rice showed that the grain number per panicle of *LAX1* haplotype is significantly higher in *indica* than in *japonica* (Wei et al. 2021)*.* Genome sequencing analysis of an allelic mutant of *LAX1* revealed that *japonica* haplotype mutates into the *indica* haplotype of *LAX1* and exhibits *lax* characteristics (Mohammad and Sang., 2012). Therefore, these results indicate that other loci (besides *the LAX1* gene) may be involved in the panicle morphogenesis of *indica* rice.

In the present study, *Oryza sativa Disproportionating Enzyme 2* (*OsDPE2*) gene in *Oryza sativa* L. subsp. *indica* cv. Dular, could rescue the *LAX1* mutant phenotype in *Oryza sativa* L. subsp. *japonica* cv. ZH11. *OsDPE2* encoded a cytoplasmic Disproportionating Enzyme 2 involved in starch breakdown at the vegetative and reproductive growth stages of rice. OsDPE2 (AQ) also had higher functional activities than the other two haplotypes. Furthermore, results show that OsDPE2 enzyme may be associated with rice panicle yield.

## Results

### *OsDPE*2 Can Rescue Panicle Mutant Phenotype of *lax1-6* in Dular

**Fig. 1:**
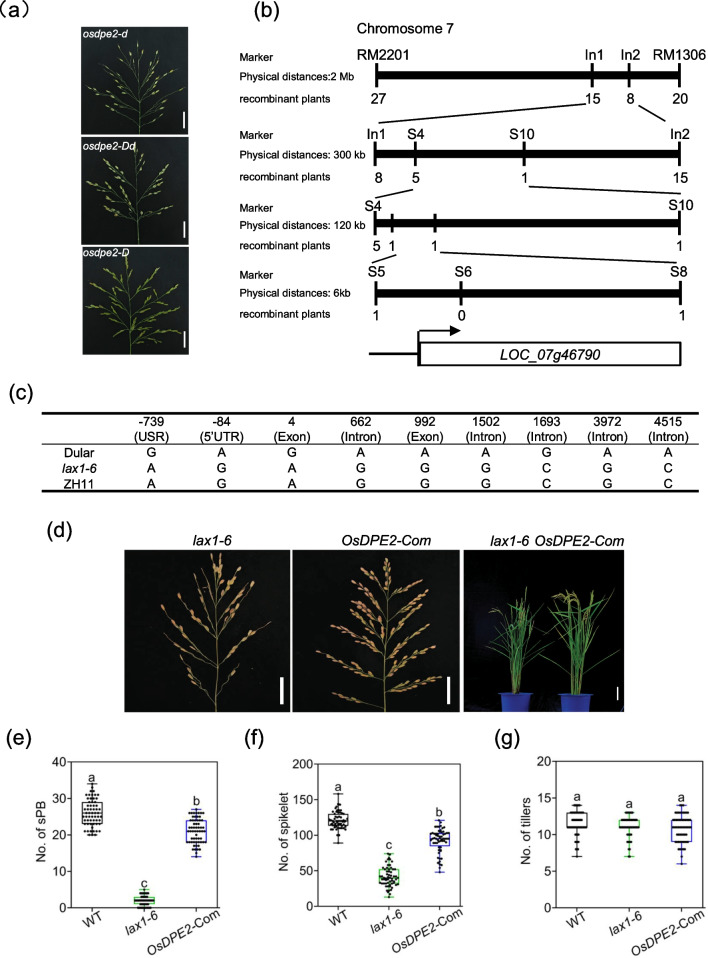
*OsDPE2* can rescue panicle mutant phenotype of *lax1-6* in Dular. **a** Panicle phenotype of *lax1-6* × Dular F2 plants with the *lax1-6* mutant genotype divided into *osdpe2-d*, *osdpe2-Dd*, and *osdpe2-D*, scale bar = 4 cm. **b** Location of *OsDPE2* via mapping. *OsDPE2* was initially mapped using *lax1-6* genotype plants of *lax1-6* × Dular F2 population. *OsDPE2* was mapped on chromosome 7 between RM2201 and RM1301 via bulked separate analysis. A total of 1600 F2 individuals screened for the *lax1-6* mutant genotype were used to fine-map *OsDPE2*. Finally, *OsDPE2* was located on chromosome 7 between Markers S5 and with only one candidate gene *LOC_07g46790*. **c** Parental comparative sequencing test for Dular, ZH11, and *lax1-6*. USR; Upstream region. **d** The number of secondary panicle branches in *OsDPE2-Com*, *lax1-6*, and wild-type. Scale bar of panicle = 4 cm; plant = 20 cm. **e**–**g** Yield traits of WT, *lax1-6*, and *OsDPE2-Com*. n = 60 biologically independent samples. Bars represent mean ± SE; Tukey's multiple comparisons test. Different letters represent statistically significant differences between data groups

Two mutant alleles of *LAX1, lax1-6,* and *lax1-3*, were identified in rice (*Oryza sativa* L. subsp. *japonica* cv. ZH11) with defective lateral branch formation by screening the *lax panicle* mutants from the T-DNA mutant library (Additional file 5: Fig. S1). The T-DNA insertion sites were found at 4905 bp and 1416 bp upstream of *the LAX1* start codon of *lax1-6* and *lax1-3, respectively* (Additional file 5: Fig. S1a). Panicle phenotype analysis showed that *lax1-6* was a weak mutant allele of *LAX1.* Unlike wild-type, *lax1-6* did not have secondary branches and significantly decreased spikelets. Further results showed that *lax1-3* was a strong mutant allele of *LAX1,* and could produce a few sterile spikelets (Additional file 5: Fig. S1b). *LAX1* gene expression analysis in 2 mm young panicles of WT, *lax1-6*, and *lax1-3* showed a significantly reduced *lax1-6* expression level (Additional file 5: Fig. S1c). The experimental rice lines were then obtained from a cross-population between two parental rice lines (*lax1-6* and Dular) to further determine whether other loci (besides *the LAX1* gene) are involved in the panicle morphogenesis of *indica* rice. Hybrid F_2_ populations were generated using *lax1-6* and Dular as the parental lines. The mutant plants were screened by identifying their *lax1-6* locus. The panicle phenotype of these plants were then observed and analyzed. Thirty plants with *the lax* phenotype (*osdpe2-d*) and 83 plants with wild-type phenotype (*osdpe2-Dd* or *osdpe2-D*) were selected for further analysis (Additional file 5: Fig. S2a). Genetic analysis of *lax1-6* × Dular F_2_ plants via Chi-Squared test showed that the *OsDPE2* locus was consistent with the dominant inheritance of a single locus (Additional file 1: Table S1). The panicle phenotype of *lax1-6* × Dular F_2_ plants was analyzed. Those with the *lax1-6* mutant genotype were divided into *osdpe2-d* (*lax1-6* type), *osdpe2-Dd* (like-wildtype), and *osdpe2-D* (wild-type) based on the ratio of secondary panicle branches/primary panicle branches (Fig. 1a).

Two SSR markers (RM2201 and RM1301), two InDel markers (In1 and In2), and five SNP markers were developed for primary mapping and fine mapping of the *lax1-6* locus (Additional file 2: Table S2). Bulked separate analysis was used to map *OsDPE2* between RM2201 and RM1301 on chromosome 7. A total of 1600 F_2_ individuals screened for the *lax1-6* mutant genotype were used to fine-map *OsDPE2*. Two recombinants were identified between S5 and S8, and the mapping area was reduced to about 6 kb with only one candidate gene (*LOC_07g46790*) (Fig. 1b). The *lax* panicle phenotype in the progenies of *OsDPE2*-d1 and *OsDPE2*-d2 were identified through genotypic identification and phenotypic observation of the recombinants identified between S5 and S8 via the progeny test (Additional file 5: Fig. S3). This result showed that *OsDPE2*-d1 and *OsDPE2*-d2 recombinants were *lax1-6* homozygotes of *OsDPE2*. Moreover, *OsDPE2*-D1, *OsDPE2*-D2, *OsDPE2*-D3, and *OsDPE2*-D4 recombinants of *OsDPE2* were Dular homozygotes because their progenies had wild-type panicle phenotypes.

Furthermore, a parental comparative sequencing test for Dular, ZH11, and *lax1-6* using S4 and S10 mapping regions was performed to identify the *OsDPE2* gene. The sequence analysis results detected nine SNPs in the S4 and S10 mapping regions of Dular, ZH11, and *lax1-6* (Fig. 1c). Among the SNPs, two non-synonymous SNPs, which can alter the amino acid coding sequence, were detected in the exon region of the genome of rice at the 4th and 992nd positions of *LOC_Os07g46790* ORF.

Complementary transgenic plants in *the lax1-6* background (*OsDPE2-*Com) were generated to verify *the OsDPE2* candidate gene. A Zhenshan 97 BAC was used to obtain 16 kb fragments containing *OsDPE2*, with no genomic sequence differences with Dular. Fragments containing *OsDPE2* were then fused with pCAMBIA2301 via one-step ligation. The progenies of the *OsDPE2-*Com T_1_ in *the lax1-6* and *lax1-3* background were subjected to phenotypic analysis and positive transgenic detection (Additional file 5: Figs. S4 and S5). *OsDPE2-*Com exhibited a partially reductive phenotype, of which some primary and secondary panicle branches retained a certain level of *lax1-6 and lax1-3*. Compared with *lax1-6*, *OsDPE2-*Com had a significantly increased number of secondary panicle branches closer to those of the wild-type (Fig. 1d–g). Therefore, the candidate gene *LOC_Os07g46790* was *OsDPE2.*

### *OsDPE2* Encodes Rice Disproportionating Enzyme 2 Located in the Cytoplasm

**Fig. 2:**
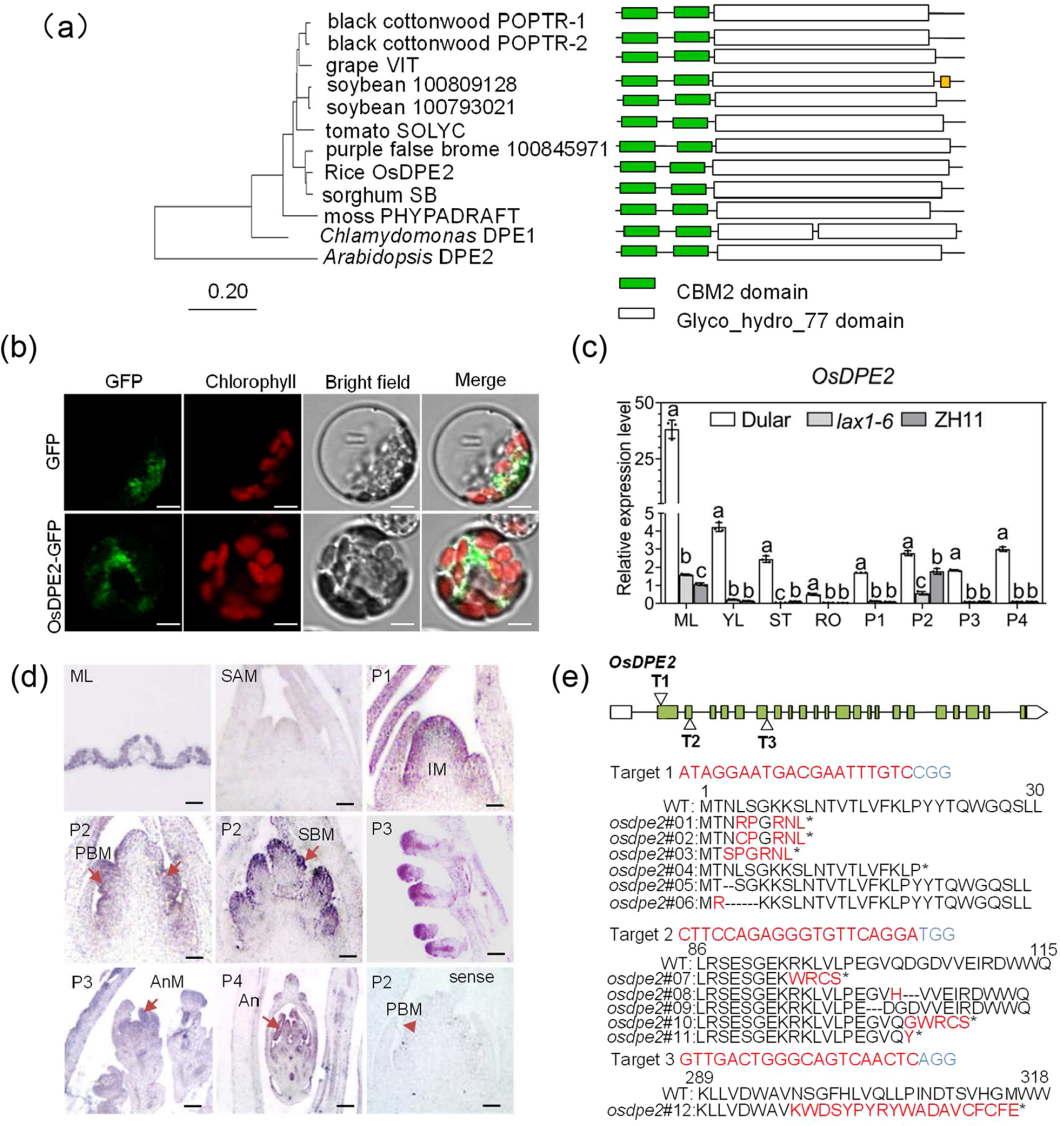
*OsDPE2* encodes rice Disproportionating Enzyme 2 located in the cytoplasm. **a** Phylogenetic analyses of DPE2 protein sequences from rice, *Arabidopsis*, soybean, tomato, sorghum, black cottonwood, grapes, purple false brome, moss, and *Chlamydomonas*. **b** Subcellular localization analysis of OsDPE2 in rice. OsDPE2-GFP and control GFP were transiently transformed into rice protoplasts. OsDPE2-GFP fluorescence signal was mainly detected in cytoplasm. Scale bar = 5 μm. **c** RT-qPCR showing the expression pattern of *OsDPE2* in the mature young leaf (YL), stem (ST), root (RO), and in several developmental stages of young panicles (1 mm (P1), 2 mm (P2), 3–5 mm (P3), and > 10 mm (P4) young panicles) in ZH11, *lax1-6* and Dular; Tukey's multiple comparisons test. Different letters represent statistically significant differences between data groups. **d** *OsDPE2*-specific antisense chain probes used for in situ hybridization of the paraffin sections of leaves and young panicles at various stages of ZH11 background samples. *SAM* secondary apical meristem, *IM* inflorescence meristem, *PBM* primary branch meristem, *SBM* secondary branch meristem, *SM* spikelet meristem, *AnM* anther meristem, *An* anther, *T* tapetum. Scale bar = 50 μm. **e** Twelve allelic mutants from transgenic ZH11 plants. *osdpe2#01* was used for subsequent functional analysis in ZH11

*OsDPE2* encodes rice DPE2, whose protein sequence was obtained from the Rice Genome Annotation Project (RGAP). The protein sequence of DPE2 was analyzed using SMART software, and results showed that OsDPE2 has two carbohydrate-binding modules (CBM-2) and a glycoside hydrolase family 77 (Glyco-hydro-77). Phylogenetic analyses of DPE2 protein sequences from rice, *Arabidopsis*, soybean, tomato, sorghum, black cottonwood, grapes, purple false brome, moss, and *Chlamydomonas* were conducted to examine the evolutionary characteristics of the DPE2 protein family (Fig. 2a). TAIR prediction showed that OsDPE2 protein could be located in the cytoplasm in Arabidopsis. The OsDPE2 coding sequence fused with a green fluorescent protein (GFP) was transfected into rice protoplasts from etiolated seedlings to verify the above. Results showed that OsDPE2::GFP proteins were located in the cytoplasm (Fig. 2b), suggesting that OsDPE2 protein is located in the cytoplasm in rice. The expression pattern of OsDPE2 in the mature leaf (ML), young leaf (YL), stem (ST), root (RO), and in several developmental stages of young panicles (1 mm (P1), 2 mm (P2), 3–5 mm (P3), and > 10 mm (P4) young panicles) was analyzed via RT-qPCR to understand the spatiotemporal expression of *OsDPE2* among Dular, *lax1-6* and ZH11 (Fig. 2c). *OsDPE2* was constitutively expressed in Dular, and its highest expression was detected in the leaves and 2 mm panicles of ZH11 and *lax1-6* background.

Furthermore, *OsDPE2*-specific antisense chain probes were designed for in situ hybridization of the paraffin sections of leaves and young panicles at various stages of ZH11 background samples. Hybridization signals were detected in mesophyll cells and the outer cells at the initial stage of inflorescence meristem (P1). The hybridization signals were also found in the outer cells at the initial stage of the primary branch meristem (P2). Hybridization signals were also detected in the outer cells of the secondary branch meristem (P2) and spikelet meristem at the initial stage. Additionally, hybridization signals were detected in the outer cells of anther meristem at the initial stage of spikelet meristem (P3) and in the outer cells of the anthers and glume at the spikelet maturation stage (P4) (Fig. 2d).

*OsDPE2* gene-editing transgenic plants in Dular were generated via CRISPR to assess the role of OsDPE2 in rice. However, homozygous mutant plants were not obtained except for one heterozygous plant (*osdpe2*^Dular^(H)). *osdpe2*^*Dular*^(H) plants exhibited short panicles, infertility, and dwarfism (Additional file 5: Fig. S6). Moreover, 12 allelic mutants were screened from transgenic ZH11 plants containing the Cas9 target site sequence. The edited amino acid sequences of the 12 allelic mutants (Fig. 2e) were identified via Sanger sequence analysis. The DNA sequence was used to predict the amino acid sequence. *osdpe2*#01 was used for subsequent functional analysis in ZH11.

### OsDPE2 Affects Vegetative Plant Development of Rice via DPE2 Enzyme Activity

In this study, WT and *osdpe2*#01 plants were grown under continuous light (CL) and continuous dark (CD) to assess whether OsDPE2 may affect vegetative plant development. Although the growth of *osdpe2*#01 was significantly inhibited under CD, *osdpe2*#01 and WT had a similar growth rate under CL (Additional file 5: Fig. S7a, b). An in-gel DPE2 enzyme activity assay was used to further analyze DPE2 enzyme activity in the WT plants under CL and CD, and OD values of the DPE2 enzyme activity. The results revealed that the DPE2 enzyme activity level had a transitory increase associated with the continuous decline in CL condition and a continuous increase associated with the transitory decline in CD (Additional file 5: Fig. S7c, d).

**Fig. 3:**
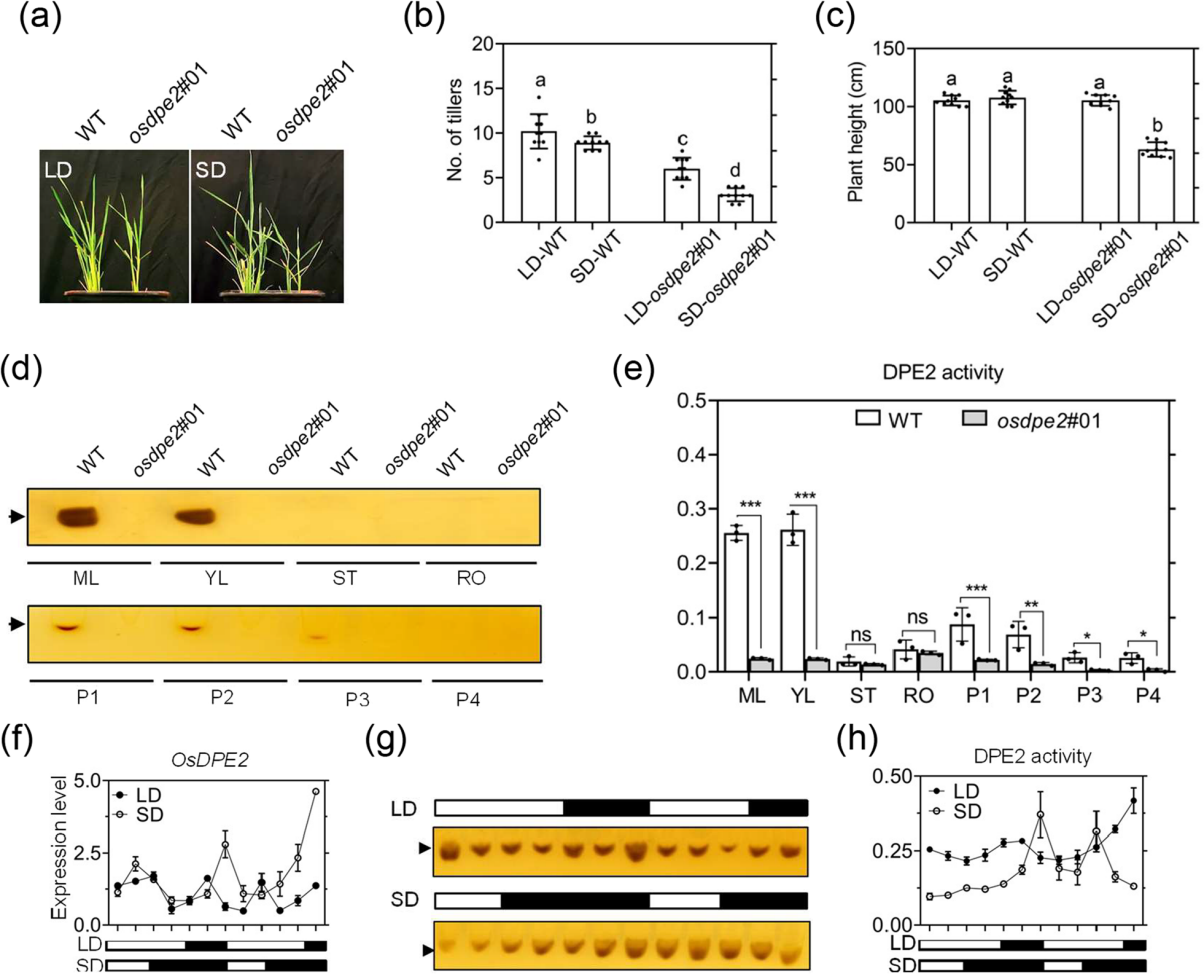
OsDPE2 affects vegetative plant development of rice via DPE2 enzyme activity, and DPE2 enzyme activity fluctuates rhythmically due to diurnal alternations. **a**–**c** Growth rate analysis of the plants under long day (LD, 16 h light/8 h dark) and short day (SD, 8 light/16 dark) conditions. **a** Images of wild-type and *osdpe2#01* seedlings under LD and SD. Number of tillers (**b**) and plant height (**c**) of wild-type and *osdpe2#01* seedlings under LD and SD conditions. Data are expressed as mean ± SD (n = 10 biologically independent samples). Bars represent mean ± standard deviation; Tukey's multiple comparisons test. Different letters represent statistically significant differences between data groups. **d** DPE2 enzyme activity and **e** OD values in ML, YL, ST, RO, P1, P2, P3, and P4. Three samples of ML, YL, ST and RO were mixed to form a biological replicate, and each data group contained three biological replicates. One biological replicate contained ten young panicle tissues. Bars represent mean ± SE (one-way ANOVA). **f** The 48-h rhythmic *OsDPE2* expression analysis of the WT plants under LD and SD conditions. **g** The 48-h rhythmic in-gel DPE2 enzyme activity assay and **h** OD values of the DPE2 enzyme activity assay of WT under LD and SD conditions. The arrow indicates DPE2 enzyme activity

Four-leaved stage seedlings of WT and *osdpe2*#01 were transplanted under long day (LD, 16 h light/8 h dark) and short day (SD, 8 light/16 dark) conditions to verify these results. The growth rate of the plants was then analyzed after 30 days. The growth of *osdpe2*#01 was significantly inhibited under SD, but *osdpe2*#01 and WT had a similar growth rate under LD (Fig. 3a–c).

An in-gel DPE2 enzyme activity assay was used to evaluate DPE2 enzyme activity and OD values of the DPE2 enzyme activity to assess whether OsDPE2 protein can form a heteroglycan-enzyme complex and transfer the heteroglycan to the non-reducing end of maltose. The extracts from mature leaf (ML), young leaf (YL), stem (ST), root (RO), and various panicle sections (1 mm (P1), 2 mm (P2), 3–5 mm (P3). and > 10 mm (P4) young panicles) of WT and *osdpe2*#01 plants were used for the assay. DPE2 enzyme activity was increased in ML, YL, P1, and P2 of WT, while DPE2 enzyme activity was not detected in *osdpe2*#01 (Fig. 3d, e). Additionally, a 48-h rhythmic expression analysis of the WT plants under LD and SD conditions was conducted to determine whether *OsDPE2* expression is associated with plant growth under light or dark conditions. The results showed that the expression level of *OsDPE2* had rhythmic oscillation with day and night alternation in LD and SD conditions (Fig. 3f). Further analyses revealed that the expression of *OsDPE2* exhibited *a* transitory increase associated with the continuous decline in the light and a continuous increase associated with the transitory decline in the dark. Meanwhile, a 48-h rhythmic DPE2 enzyme activity analysis of WT and *osdpe2*#01 under LD and SD conditions showed that OsDPE2 enzyme activity also had a rhythmic oscillation with day and night alternation in LD and SD conditions. Similarly, OsDPE2 enzyme activity also showed a transitory increase associated with the continuous decline in the light and a continuous increase associated with the transitory decline in the dark (Fig. 3g, h). In summary, OsDPE2 affects the vegetative plant development of rice via DPE2 enzyme activity. Furthermore, DPE2 enzyme activity fluctuates rhythmically due to diurnal alternations.

### *OsDPE2* Affects Panicle Morphogenesis in Rice

**Fig. 4:**
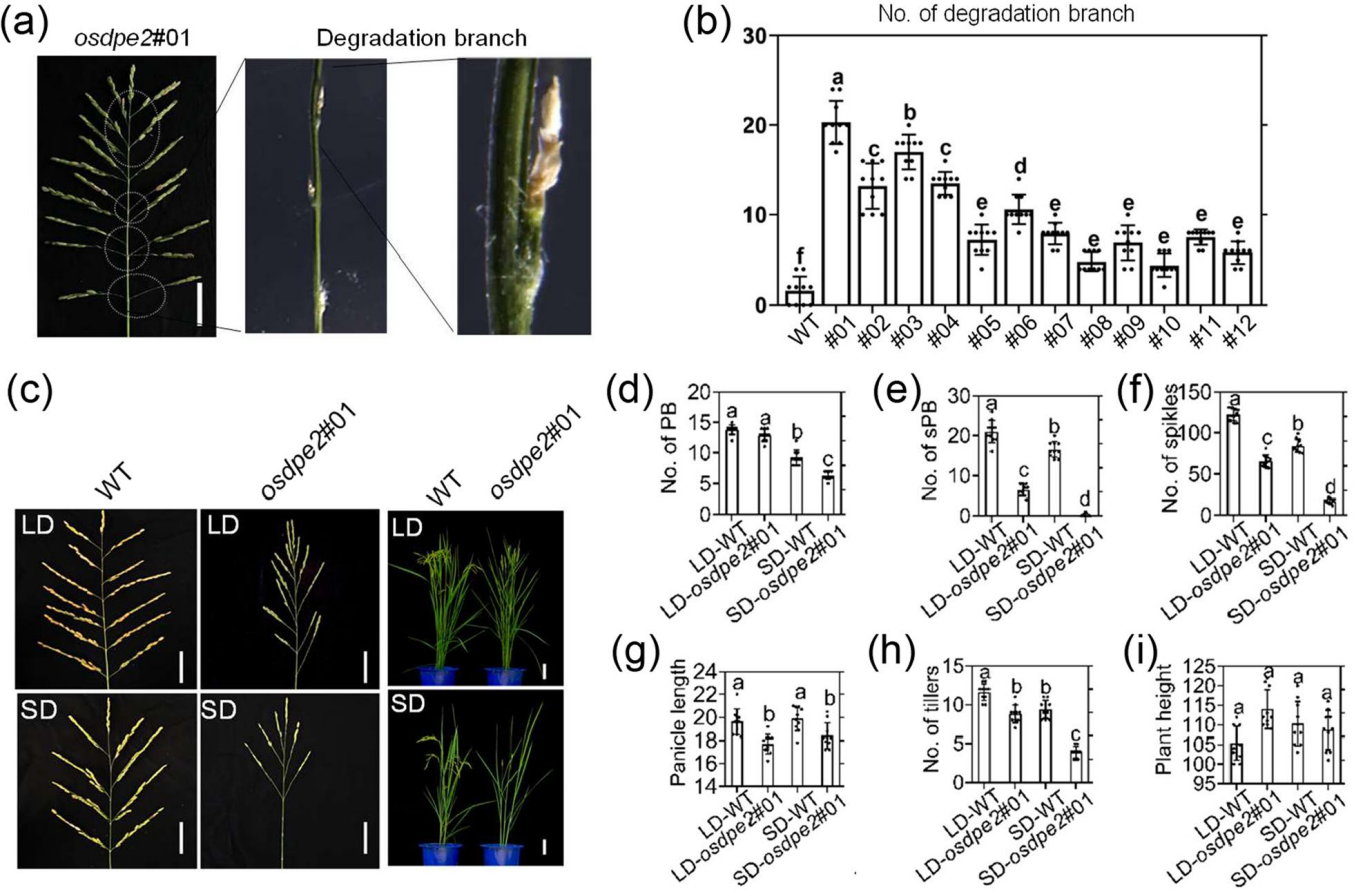
*OsDPE2* regulates panicle morphogenesis of rice. **a** Phenotypic characteristics of degradation branches in *OsDPE2* mutants. **b** Number of degradation branches of WT and 12 allelic *OsDPE2* mutants. **c** Comparative analysis of panicle and plant phenotypic characteristics. **d**–**i** Yield trait of WT and *osdpe2#01* under LD and SD conditions. Scale bar of panicle = 4 cm and plant = 20 cm. n = 10 biologically independent samples

Compared with the WT plants, the degradation branches significantly increased in OsDPE2 mutants. Furthermore, protein variation caused by editing was correlated with the number of degenerated branches (Fig. 4a, b; Additional file 5: Fig. S8). The 30-day seedlings of WT and *osdpe2*#01 plants were grown under LD and SD conditions until flowering to analyze whether OsDPE2 affects reproductive growth in rice. Comparison analysis showed that the number of secondary branches of the panicle and the number of spikelets per panicle reduced in *osdpe2*#01 plants as the sunlight duration changed from long to short (Fig. 4c). Moreover, analyses of plant and panicle yield traits showed that *osdpe2*#01 had significantly decreased primary branches of the panicle, secondary branches of the panicle, spikelets per panicle, panicle length, and tiller number, and a significant increase in plant height under LD and SD conditions compared with WT (Fig. 4d–i). Further analysis revealed that other plant traits and panicle yield traits of the WT and *osdpe2*#01 significantly decreased under SD than under LD conditions, except for the plant height. Compared with LD conditions, the number of secondary branches of the panicle, spikelets per panicle, and tiller of *osdpe2*#01 significantly decreased under SD conditions. These results indicate that *OsDPE2* affects panicle morphogenesis by regulating plant growth and development in the dark.

### *OsDPE2* Regulates Panicle Morphogenesis by Affecting Starch Content

**Fig. 5:**
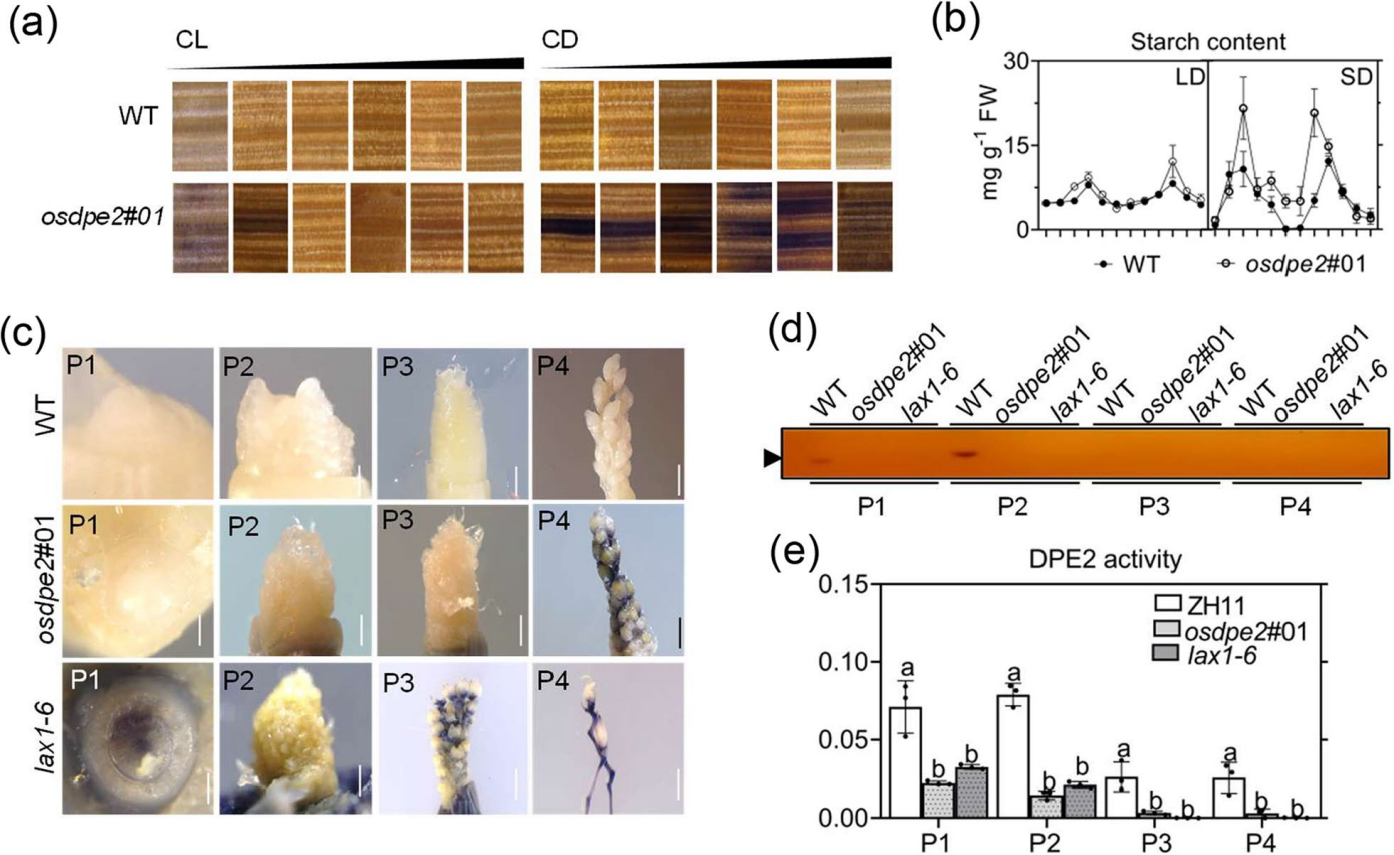
*OsDPE2* affects panicle morphogenesis by regulating starch content. **a** Iodine staining analysis of mature leaves under continuous light (CL) and continuous dark (CD) conditions. We treated the seedlings under CL and CD for 2, 4, 6, 8, 10, 12 h (from left to right). Three biological replicates at each time point were sampled. **b** The 48-h rhythmic starch content analysis of the WT and *osdpe2#01* plants under LD and SD conditions. Three leaf samples were mixed into a biological replicate at each time point, and each data group had three biological replicates. Bars represent mean ± SE. **c** Iodine staining analysis at various young panicle stages of WT, *osdpe2#01*, and *lax1-6*. Scale bar = 50 μm. Phenotypic characteristics of degradation branches in *OsDPE2* mutants. **d** In-gel DPE2 enzyme assay and **e** OD values of the DPE2 enzyme assay of P1, P2, P3, and P4 of ZH11, *osdpe2#01*, and *lax1-6*. Twenty young panicle samples were mixed into a biological replicate at each time point, and each data group had three biological replicates. Bars represent mean ± SE; Tukey's multiple comparisons test. Different letters represent statistically significant differences between data groups

Iodine staining analysis of starch in various vegetative organs of wild-type rice showed that ML and YL of *osdpe2*#01 had stronger staining signals than the wild-type (Additional file 5: Fig. S9). Additionally, Iodine staining analysis of ML under CL and CD conditions revealed that the starch content was significantly higher in *osdpe2*#01 than in WT under CD conditions (Fig. 5a). Furthermore, a 48-h rhythmic starch content analysis of the WT and *osdpe2*#01 plants was conducted under LD and SD to determine whether starch content is associated with OsDPE2 enzyme activity. Compared with WT in the dark, starch content significantly increased in *osdpe2*#01 plants (Fig. 5b), indicating that OsDPE2 may be involved in starch breakdown during the vegetative growth of rice.

Iodine staining analysis at the various young panicle stages of WT, *osdpe2*#01, and *lax1-6* was performed to establish whether OsDPE2 plays a similar role in starch metabolism during rice growth. The results revealed that young panicles of *osdpe2*#01 and *lax1-6* had a stronger staining signal than WT, suggesting that OsDPE2 may be involved in starch breakdown during the development stages of young rice panicles (Fig. 5c). Further analysis did not detect DPE2 enzyme activity in various young panicle stages of the WT, *osdpe2*#01, and *lax1-6 plants,* similar to *osdpe2*#01 (Fig. 5d, e). These results imply that *OsDPE2* regulates panicle morphogenesis of rice by affecting starch content.

### Haplotype OsDPE2 (AQ) with Higher DPE2 Enzyme Activity Increases Panicle Yield of Rice

**Fig. 6:**
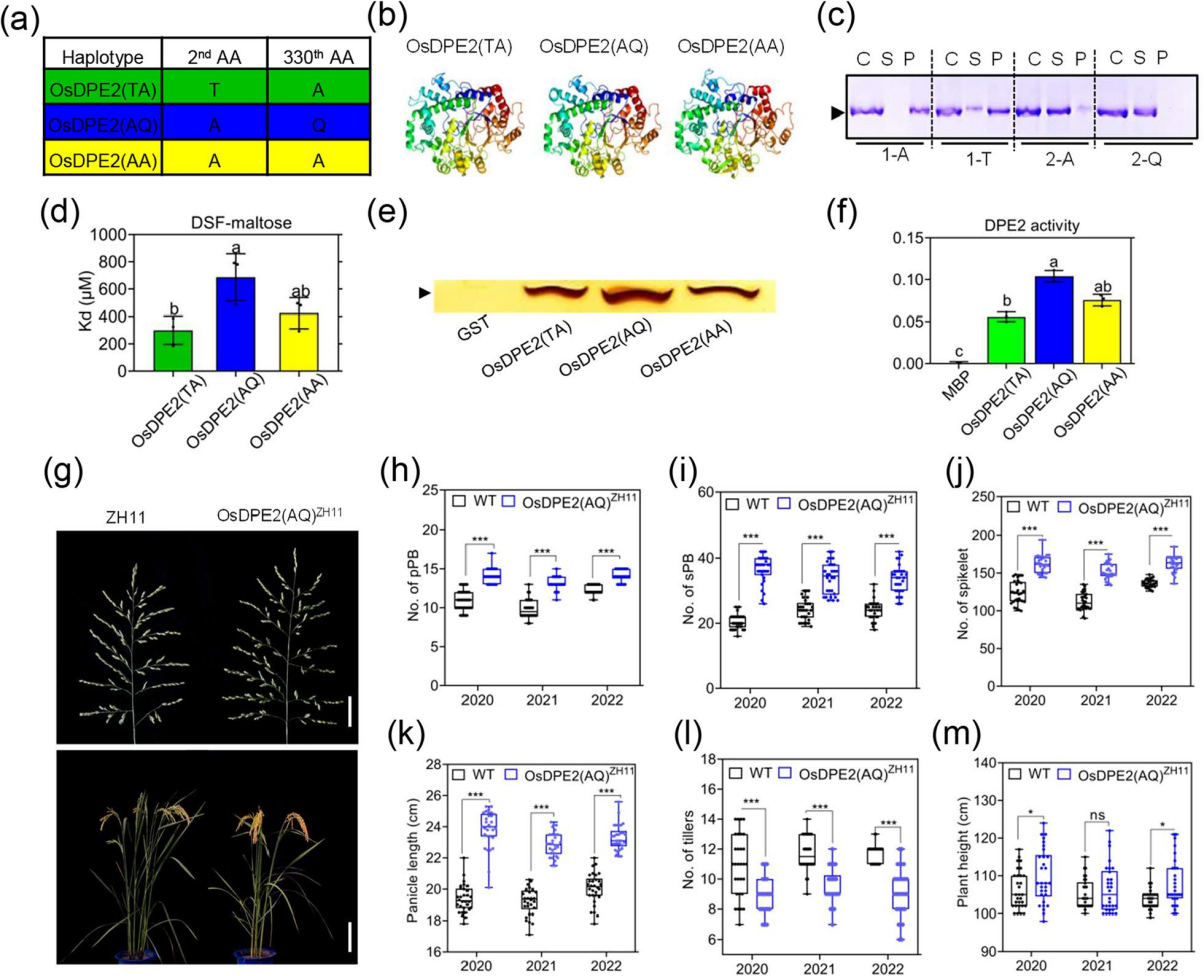
Haplotype OsDPE2 with higher DPE2 enzyme increases the panicle yield of rice. **a** Haplotype analysis showing the 2nd and 330th amino acids of the *OsDPE2* divided into three haplotypes: OsDPE2(TA), OsDPE2(AQ), and OsDPE2(AA). **b** Protein spatial structure of OsDPE2 haplotypes predicted by Phyre2. **c** Analysis of starch binding capacity of different haplotypes of OsDPE2 proteins and different CBM2 domain proteins with MBP tag. Starch binding capacity was determined as previously described (Ruzanski et al; 2013). Briefly, starch (20 mg) was incubated with label protein (20 μM) at 4 ℃for 30 min. The reaction system was divided into precipitate (P) and supernatant (S) via centrifugation. The gel underwent vertical electrophoresis with 8% protein PAGE gel after denaturing the same amount of control group (C), supernatant (S) and precipitate (P). **d** The binding of recombinant proteins to the maltose assay of OsDPE2 haplotypes via differential scanning fluorimetry (DSF). Each data group had three replicates; Tukey's multiple comparisons test. Different letters represent statistically significant differences between data groups. **e** In-gel DPE2 enzyme assay and **f** OD values of OsDPE2 haplotypes. Each data group had three replicates; Tukey's multiple comparisons test. Different letters represent statistically significant differences between data groups. **g** Panicle and plant phenotypic characteristics of ZH11 and OsDPE2 (AQ)^ZH11^. Scale bar of panicle = 4 cm and plant = 20 cm. **h**–**m** Yield traits of WT and OsDPE2(AQ)^ZH11^ for three consecutive years. Sidak's multiple comparisons test. Bars represent mean ± SE (one-way ANOVA). n = 30 biologically independent samples

Information of genomic variations of Single nucleotide polymorphism (SNP) of 504 cultivated rice accessions from Ricevarmap databases (Zhao et al. 2015) were used to analyze the haplotypes and their evolution at the *OsDPE2* locus. The results revealed that the SNPs located at the 4th and 992nd nucleotide of the *OsDPE2* CDS were divided into three haplotypes: OsDPE2(TA), OsDPE2(AQ), and OsDPE2(AA). OsDPE2 (AA) is mainly distributed in *Temperate japoni*ca (55.91%), while OsDPE2 (AQ) was mainly distributed in *indica* (53.85%), *aus* (15.22%) and *Intermedia rice* (50%). OsDPE2(TA) was mainly found in *Temperate japonica* (40.36%), *Tropical japonica* (90.70%), *indica* (42.86%), *aus* (84.78%), and *intermedia* (46.43%) (Fig. 6a; Additional file 5: Fig. S10a). Furthermore, Phyre2 showed that the spatial protein structures of OsDPE2 haplotypes were different, possibly because of amino acid sequence variation (Fig. 6b). Phylogenetic analyses of *OsDPE2* in the OsDPE2 haplotypes, wild rice, and other rice germplasm resources were conducted using EggNOG v5.0 software to uncover the evolution of OsDPE2 haplotypes (Huerta et al. 2019). The result revealed that *O. rufipogon* was closely related to OsDPE2(AA), OsDPE2(AQ), and OsDPE2(TA) (Additional file 5: Fig. S10b).

A starch assay was performed to assess whether OsDPE2 haplotypes have functional variations. The recombinant proteins were bound to OsDPE2 haplotypes and the CBM2 domain. Results showed that there were no significant functional variations among OsDPE2-GST haplotypes. However, CBM2-1(A) had a higher binding affinity than CBM2-1(T) but lower than that of CBM2-2 (Q) (Fig. 6c; Additional file 5: Fig. S11). The binding of recombinant proteins to the OsDPE2 haplotypes was also conducted using differential scanning fluorimetry (DSF) to distinguish the functional strength of the OsDPE2 haplotypes. The Melting temperature (℃) of each OsDPE2-GST haplotype increased logarithmically with the maltose concentration from 100 to 1000 mM (Additional file 5: Fig. S12). Furthermore, OsDPE2 (AQ) had a significantly higher binding ability than the other two haplotypes (Fig. 6d). Similarly, OsDPE2 (AQ)-GST had a significantly higher DPE2 enzyme activity than the other two haplotypes (Fig. 6e, f). These results suggested that OsDPE2 (AQ) can function better than the other haplotypes.

Therefore, OsDPE2 haplotypes may have evolved with rice domestication and can affect rice panicle yield. A transgenic plant line, OsDPE2(AQ)^ZH11^ was generated using the OsDPE2 (AQ) sequence of Dular ligated to pC2301 vector skeleton, which was transformed into ZH11(haplotype of OsDPE2 is OsDPE2(TA)), to verify the above assumption. Six transgenic lines containing single copies were screened from T_0_ generation via quantitative multiplex real-time PCR (Additional file 5: Fig. S13; Additional file 3: Table S3). The isolated population plants were used to investigate the yield traits after one generation of self-crossing. The panicle yield characteristic of OsDPE2 (AQ)^ZH11^ and ZH11 was then analyzed for three consecutive years. The results showed that OsDPE2 (AQ)^ZH11^ had more panicles and more panicle branches and spikelets per panicle than ZH11 (Fig. 6g–m), indicating that haplotype OsDPE2 (AQ) can increase the panicle yield of rice.

## Discussion

**Fig. 7:**
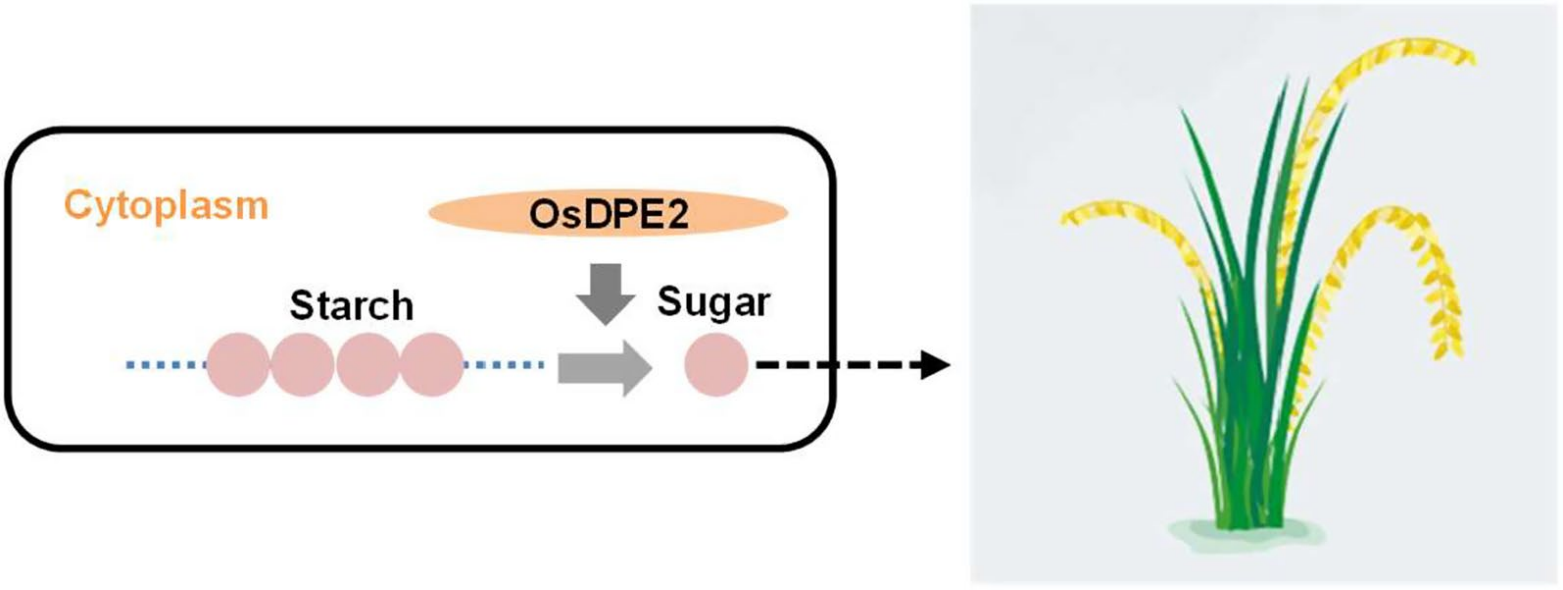
Model of *OsDPE2* regulating rice panicle morphogenesis. OsDPE2participates in starch breakdown, thus providing available carbon source for vegetative and reproductive plant development of rice

Starch transitions from a carbon sink during the day to a carbon source at night in vegetative tissues (MacNeill et al. 2017). Fixed carbon dioxide, in the form of soluble sugars, is metabolized to glucose and short amylose during the day, which are transported to heterotrophic tissues, such as roots or immature leaves, as sucrose or stored as starch to provide energy for growth. The starch is consumed at night to provide carbon source for plant growth. In this study, results showed that *OsDPE2* gene participates in starch breakdown, thus providing available carbon source for vegetative and reproductive development of rice (Fig. 7).

Furthermore, *OsDPE2* could rescue the mutant phenotype of *lax1-6*. This could be because; 1) OsDPE2 can act as the upstream regulator of *LAX1* to modulate the expression *of LAX1*, and 2) LAX1 can act as the upstream regulator of *OsDPE2* to modulate the expression of *OsDPE2*. Comparative sequencing analysis detected a non-synonymous variation at the 4th (CBM2-1 domain) and 992nd (CBM2-2 domain) nucleotide of *OsDPE2* ORF in ZH11, *lax1-6*, and Dular. This can lead to variation in the amino acid sequence, resulting in protein variation. Further analyses proved that OsDPE2 (AQ), distributed to found in Dular, had higher DPE2 enzyme activity (Fig. 6). Meanwhile, RT-qPCR analysis indicated that *LAX1* affected *OsDPE2* expression in 2 mm young panicle in the ZH11 and *lax1-6* plants. These results support the above assumption. Therefore, LAX1 can regulate *OsDPE2* expression by acting as the upstream regulator of *OsDPE2*. Nevertheless, further studies are needed to support this conclusion.

As signal factors, sugars also regulate plant inflorescence growth (Lastdrager et al. 2014; Li and Sheen 2016). In maize, a trehalose-6-phosphate phosphatase encoded by *RAMOSA3* (*RA3*) is expressed in discrete domains subtending axillary inflorescence meristems (Satoh-Nagasawa et al. 2006). Furthermore, the meristem determinacy regulated by trehalose 6-phosphate phosphatases is uncoupled from enzymatic activity (Claeys et al. 2019). Moreover, arabinosylated CLV3 can rescue the tomato inflorescence and branching mutants with defective arabinosyltransferase genes (Fletcher et al. 1999; Xu et al. 2015). *OsDPE2* loss-function mutants in rice have reduced panicle branches and spikelets at the reproductive stage than the WT under LD and SD conditions (Fig. 4), especially under SD conditions. In this study, starch content of reproductive tissues of WT, *osdpe2*#01, and *lax1-6* was negatively correlated with DPE2 enzyme activity and panicle yield. Therefore, the loss of OsDPE2 function in the vegetative and reproductive tissues of rice can inhibit starch transfer or metabolism, while excessive storage of non-soluble sugars (starch) can inhibit plant growth. However, further studies should assess whether sugar signal factors, which act downstream of OsDPE2, can also regulate the growth and development of specific tissues and organs of rice.

Starch can be used as either a carbon source or sink, depending on the temporal and spatial context. Moreover, the changing role depends on the interaction between genetic and metabolic functions that determine the timing and transformation of the developmental process. Plants are conservative in energy use and store available organic matter before flowering. This is because plants use this surplus energy to produce viable seeds when it enters the reproductive stage only when sufficient energy is stored in the vegetative tissues (Yu et al. 2000). Plants can also change the source-sink ratio under environmental change or stress, depending on the developmental stage. For example, plants may reduce the transport of organic matter to seeds in the early reproductive development by aborting the early reproductive tissue (pollen grains or ovule), thus reducing the sink strength and maternal organic matter output (Sun et al. 2004). In this study, the growth of *osdpe2*#01 was significantly inhibited under CD and SD compared with the wild-type. Panicle yield of *osdpe2*#01 exhibited the characteristics of the *lax* and short panicle under SD condition after entering the reproductive growth stage. These results indicate that OsDPE2 can maintain normal growth and development of plants in dark, showing that plant produces viable seeds when it enters the reproductive stage.

The genetic basis underlying the determinants of rice panicle yield has been studied by mapping their associated quantitative trait loci (QTLs) between *indica* and *japonica* rice (Jing et al. 2018; Li et al. 2020; Qiu et al. 2012; Zhang et al. 2015). In this study, *OsDPE2* was constitutively expressed in Dular but preferentially expressed in the leaves and 2 mm panicles of the ZH11 background (Fig. 2c). However, the OsDPE2 mutant was not detected in Dular. These results indicate that OsDPE2 participates in seed germination or pollen development of Dular, showing that OsDPE2 may play different roles in *japonica* and *indica* rice. Nevertheless, further research should assess the different roles of OsDPE2 in *japonica* and *indica* rice.

## Conclusions

In summary, the identified *Oryza sativa Disproportionating Enzyme 2* (*OsDPE2*) is involved in rice panicle morphogenesis. OsDPE2 participates in starch degradation during the entire growth period of rice. DPE2 enzyme activity of OsDPE2 protein is associated with rice yield traits. Therefore, this study guides for rice breeding to improve panicle yield traits.

## Materials and Methods

### Plant Materials and Growth Conditions

T-DNA insertion mutant, *lax1-6* mutant (wild-type; cultivar Zhonghua11 (ZH11)) was sourced from the National Key Laboratory of Crop Genetic Improvement at Huazhong Agriculture University, Wuhan, China (Wu et al. 2003). *OsDPE2-*Com was generated using *lax1-6* mutant via transgenic technology. Moreover, *OsDPE2* was mapped using the most cultivated rice cultivar (*Oryza sativa subsp indica* var Dular), while *osdpe2*#01–#12 was produced using a different cultivar (*Oryza sativa subsp japonica* var ZH11)via CRISPR/Cas9 technology, as previously described (Ma et al. 2015).

Rice plants were grown in the experimental fields in Wuhan, China, from May to October. The day length in Wuhan from mid-May to early August was > 13.5 h (long days) and < 13.5 h from August to October (short days), with an average daily temperature of 26 ℃. The plants were grown in a growth chamber with 26 °C/26 °C (day/night) and relative humidity of 60% for the continuous light (CL)/continuous dark (CD) treatment experiment. However, the plants were grown in a growth chamber with 32 °C/28 °C (day/night), relative humidity of 60%, and a diurnal cycle of 16 h/8 h or 8 h/16 h (light/dark) for the long (LD) and short (SD) day treatment experiment. Fluorescent white-light tubes (400 to 700 nm, 250 mmol m^−2^ s^−1^) were used for lighting.

### Fine Mapping and Cloning

A total of 1600 F_2_ individuals screened for the *lax1-6* mutant genotype were used to fine-map *OsDPE2*. InDel markers and markers for primary and fine mapping were developed using the Ricevarmap database (http://ricevarmap.ncpgr.cn). The progeny test was also performed for the recombinant *lax1-6* × Dular F_4_ plants. The *OsDPE2* genotype was identified using the functional marker, In1.

### Vector Construction, Transformation, and Transgenic Plant Generation

Primers used are shown in Additional file 4: Table S4. A Zhenshan 97 BAC was used to generate 16 kb fragments containing *OsDPE2*, with no genomic sequence differences with Dular. The fragments containing *OsDPE2* were cloned into the binary pCAMBIA2301 vector with G418 resistance via one-step ligation of multi-fragments. The constructs were then introduced into *lax1-6* via Agrobacterium-mediated transformation, as previously described (Lin and Zhang 2005). The T_1_ families, *lax1-6* and wild-type ZH11, used as controls, were sown in a field bed in Wuhan, China.

For the gene-edited knockout of *osdpe2*#01–#12, the target genomic sequence of *OsDPE2* was designed using the CRISPR primer Designer software (http://www.plantsignal.cn) and synthesized via GenScript. The single guide RNA-Cas9 plant expression vectors were also constructed, as described by Ma (2015).

The constructs were verified via sequencing and transformed into the calli of rice cultivar ZH11, *lax1-6*, or Dular via the Agrobacterium-mediated method. Total genomic DNA was isolated from the leaves of the regenerated plants and subjected to PCR assays and sequencing to identify the positive transgenic rice plants (at least five independent lines) and mutants. Morphological and panicle yield traits in the T_3_ generation of the transgenic rice plants and the T_2_ generation of the mutants were then investigated.

### Phylogenetic Analyses

Phylogenetic analyses of DPE2 protein sequences from rice (UniProt ID: Q69Q02), Arabidopsis (UniProt ID: Q8RXD9), soybean (UniProt ID: I1JMZ7; I1N8M8), tomato (UniProt ID: A0A1S4BU65), sorghum (UniProt ID: A0A1W0W7T6), black cottonwood (UniProt ID: A0A2K1ZW06; A0A2K1X9U9), grapes (UniProt ID: F6HQQ7), purple false brome(UniProt ID: I1GRF8), moss(UniProt ID: A0A2K1JEK9), and Chlamydomonas (UniProt ID: A0A2K3E1T5) were conducted to examine the evolutionary characteristics of the DPE2 protein family.

The phylogenetic trees were constructed using the Neighbor-Joining method (Saitou and Nei 1987). The tree was drawn to scale, with branch lengths having the same units as the evolutionary distance used to construct the phylogenetic tree. The Poisson correction method (Zuckerkandl and Pauling 1965) was used to determine the evolutionary distances, which were presented in the unit number of amino acid substitutions per site. MEGA7 (Kumar et al. 2016) was used for phylogenetic analyses.

### Histological Analysis

*In-situ* hybridization was conducted as described by Hirose (2002). Briefly, young panicles were collected from cultivar ZH11 from the tillering stage to the pre-flowering stage. Probe primers used in *in-situ* hybridization are shown in Additional file 2: Table S2. Images were captured under the Nikon luminescence microscope NI-E using a DS-Ri2 camera.

Furthermore, iodine staining analysis of starch was performed using the I_2_-KI solution, as described by Li (2011). The mature leaf tissue was decolorized with absolute ethanol, and the images were captured under the Nikon stereomicroscope SMZ25 using a DS-Ri2 camera.

### Quantitative Reverse Transcription PCR (RT-qPCR)

RT-qPCR analysis was performed using RNA samples from three biological replicates unless stated otherwise. Briefly, total RNA was extracted using Trizal Kits (TransGen Biotech) and treated with RNase-free DNaseI. The DNaseI-treated RNA (1 mg) was then used to synthesize the first-strand cDNA using the enzyme RTM-MLV (Roche). Applied Biosystems ViiA 7 machine was then used for qPCR via SYBR Green Real-Time PCR Master Mix (Roche). The expression levels were analyzed via the comparative critical threshold method with rice gene *OsACTIN1* (*LOC_Os03g50885*) as the internal control, as previously reported (Caldana et al. 2007*)*.

### Subcellular Localization Analysis

The sequence encoding *OsDPE2* in ZH11 was fused to the pM999-GFP vector. The fusion protein inserted in the correct direction was transfected into rice protoplasts as previously described (Zhou et al. 2009) with minor modifications. The transformed cells were incubated in the dark at 28 °C for 20 h, then a confocal laser scanning microscope (Leica, Germany) was used to evaluate the subcellular localization of these proteins based on the fluorescence images.

### DPE2 Enzyme Activity Assay

In-gel DPE2 enzyme activity assay was performed as previously described (Critchley et al. 2001). Total protein was extracted using extraction buffer containing 100 mM 3-(N-morpholino) propanesulphonic acid (MOPS) (pH 7), 10% (v/v) glycerol, 1 mM dithiothreitol (DTT), and 4% (w/v) polyvinylpolypyrrolidone at 0–4 °C. The resolving gel contained 8% (w/v) acrylamide/bisacrylamide (30:1), 375-mM Tris–HCl, and 1% (w/v) oyster glycogen and had a pH of 8.8. The stacking gel contained 4% (w/v) acrylamide/bisacrylamide (30:1) and 63-mM Tris–HCl and had a pH of 6.8. The gel underwent electrophoresis at 12 mA and 4 °C, then washed twice with 50 mL of 100 mM Tris, 1 mM MgCl2, 1 mM ethylenediamine tetraacetic acid (EDTA), and 1 mM DTT for 10 min. The gel was then incubated with 5 mM maltose in wash buffer at 37 °C overnight. The gel was stained with 0.67% (w/v) I_2_ and 3.33% (w/v) KI. The optical density (OD) values of DPE2 enzyme activity from the extracts of all tissue types prepared by centrifugation at 15 000 g for 10 min were determined as previously described (Chia et al. 2004). The above assays (in-gel DPE2 enzyme activity assay and OD values determination) had three independent replicates.

### Extraction and Measurements of Starch

Three plant leaf samples collected at each time point were pooled into a biological replicate, and each data group contained three biological replicates. The samples were placed in microfuge tubes, frozen in liquid N_2_, and used for starch and soluble sugar extraction. Starch extraction was conducted as described by Lu (2004). Starch content was determined using NADP(H)-linked assay in a microplate reader (TECAN Infinite M200).

### Expression and Purification of Recombinant OsDPE2 and CBM2 Domain

The PCR-amplified CDS of all *OsDPE2* and CBM2 domains were ligated to pGEX-4 T-1 and PMCL-C2X via homologous recombination. The plasmid vectors were then transformed into *Escherichia coli* BL21 (DE3) cells for expression. Briefly, the bacterial cells were cultured in 200 mL of fresh Luria–Bertani broth at 16 °C until OD_600_ was at ~ 0.7 when the expression was induced by 1.0 mM of isopropyl-D-thiogalactopyranoside. The recombinant GST-OsDPE2 protein was then bound to the affinity medium of GST beads (Thermo Fisher). Impurities were removed by washing the medium using a binding buffer (140 mM of NaCl, 2.7 mM of KCl, 10 mM of Na_2_HPO_4_, and 1.8 mM of KH_2_PO_4_ at pH 7.3). The GST-OsDPE2 protein (0.53 mg/200 mL) was then eluted with an elution buffer (50 mM Tris–HCl and 10 mM of reduced glutathione at pH 8.0). Conversely, the recombinant MBP-CBM2 domain was bound to the affinity medium of MBP beads (Thermo Fisher). The impurities were removed by washing with a binding buffer (140 mMof NaCl, 2.7 mM of KCl, 10 mM of Na2HPO4, and 1.8 mM of KH2PO4 at pH 7.3). An elution buffer (50 mM of Tris–HCl and 10 mM of maltose at pH 8.0) was used to elute the GST-OsDPE2 protein (0.53 mg/200 mL). All recombinant proteins were quantified by SDS-PAGE.

### Starch and Maltose Binding Capacity Assay

Starch binding capacity was analyzed as previously described (Ruzanski et al. 2013). Differential scanning fluorescence (DSF) was used to detect the maltose binding ability of the different OsDPE2-GST haplotypes. The reaction system contained the labeled proteins (50 ng), maltose gradient concentration (1–10,000 mM), and protein fluorescent stain. The reaction was completed on an Applied Biosystems ViiA 7 quantitative PCR instrument. A solution curve program with two ran temperatures (25 ℃ (+ 1℃, 5S per cycle for 75 cycles) and 90℃ (5 min, 1 cycle)) was used. The data were then exported and analyzed using the Protein Thermal Shift Software 1.4. The analyzed data were plotted and analyzed using the mapping software GraphPad Prism 8. The maltose binding ability of the different OsDPE2-GST haplotypes was analyzed with 95% CI.

### Real-Time PCR Estimates of Copy Number

Real-time PCR estimates of copy number were performed as previously described (Omar et al. 2008). A pair of primers and an internal hybridization fluorogenic TaqMan probe for detecting the endogenous *OsDPE2* and the transgene OsDPE2(AQ)^ZH11^ in transgenic citrus plants were designed for quantitative real-time PCR (Additional file 1: Table S1). Three replicates of samples from each plant were subjected to multiplex reaction. Standard curves were obtained after serial dilution of a transgenic line using Applied Biosystems ViiA 7 machine. The copy numbers of OsDPE2(AQ)^ZH11^ gene were determined by comparing the absolutely quantified OsDPE2(AQ)^ZH11^ transcripts with those of the endogenous *OsDPE2* gene according to the standard curves. Each transgenic citrus DNA sample had four replicates to correct pipetting errors. The values were averaged to obtain the starting copy numbers of the transgene or endogenous gene.

## Supplementary Information

**Additional file1. Table S1**: Primers used in this study.

**Additional file 2. Table S2**: Information of molecular markers.

**Additional file 3. Table S3**: Genetic analysis of plants of *lax1-6×* Dular F2 by Chi-Squared test.

**Additional file 4. Table S4**: Real-Time PCR Estimates of Copy Number for OsDPE2(AQ)^ZH11^ Transgene.

**Additional file 5. Fig. S1**: T-DNA mutants of *LAX1* in ZH11. **Fig. S2**: Mutant plants were screened out to observe and analyze their panicle phenotype after identifying their *lax1-6* locus. **Fig. S3**: Genotypic identification and phenotypic observation of the recombinants identified between S5 and S8 using the progeny test. **Fig. S4**: The progenies of the *OsDPE2-Com* T_1_ in the *lax1-6* background were subjected to phenotypic analysis and positive transgenic detection. **Fig. S5**: The progenies of the *OsDPE2-Com* T_1_ in the *lax1-3* background were subjected to phenotypic analysis and positive transgenic detection. **Fig. S6**: Panicle and plant phenotypic characteristics of Dular and *osdpe2*^Dular^(H). **Fig. S7**: Growth rate analysis and DPE2 enzyme activity analysis of the plants under continuous light (CL) and continuous dark (CD) conditions. **Fig. S8**: Panicle phenotypic characteristics analysis of the 12 allelic *OsDPE2* mutants. **Fig. S9**: Iodine staining analysis of starch in various vegetative organs of wild-type and *osdpe2#01*. **Fig. S10**: Haplotype evolution and evolutionary analysis of *OsDPE2* locus. **Fig. S11**: Starch assay and bound the recombinant proteins to OsDPE2 haplotypes. **Fig. S12**: The binding of recombinant proteins to the maltose assay of OsDPE2 haplotypes using differential scanning fluorimetry (DSF). **Fig. S13**: Standard curve of endogenous *OsDPE2* and transgene OsDPE2(AQ)^ZH11^ genes.

## Data Availability

All data supporting the conclusions of this article are provided within the article (and its additional files).

## Abbreviations

DPE2: Disproportionating Enzyme 2
P1: Young panicle at initial stage of inflorescence meristem
P2: Young panicle at initial stage of the primary branch meristem
P3: Young panicle at initial stage of spikelet meristem
P4: Young panicle at spikelet maturation stage
CL: Continuous light
CD: Continuous night
LD: Long-day
SD: Short-day
