# Supplementary material for: OsDPE2 Regulates Rice Panicle Morphogenesis by Modulating the Content of Starch
Source: Rice (N Y). 2023 Feb 3;16:5. doi: 10.1186/s12284-023-00618-3 (PMC9895648; doi:10.1186/s12284-023-00618-3)
Supplement: Supplementary file 5 — Additional file 5. Fig. S1: T-DNA mutants of LAX1 in ZH11. Fig. S2: Mutant plants were screened out to observe and analyze their panicle phenotype after identifying their lax1-6 locus. Fig. S3: Genotypic identification and phenotypic observation of the recombinants identified between S5 and S8 using the progeny test. Fig. S4: The progenies of the OsDPE2-Com T1 in the lax1-6 background were subjected to phenotypic analysis and positive transgenic detection. Fig. S5: The progenies of the OsDPE2-Com T1 in the lax1-3 background were subjected to phenotypic analysis and positive transgenic detection. Fig. S6: Panicle and plant phenotypic characteristics of Dular and osdpe2Dular(H). Fig. S7: Growth rate analysis and DPE2 enzyme activity analysis of the plants under continuous light (CL) and continuous dark (CD) conditions. Fig. S8: Panicle phenotypic characteristics analysis of the 12 allelic OsDPE2 mutants. Fig. S9: Iodine staining analysis of starch in various vegetative organs of wild-type and osdpe2#01. Fig. S10: Haplotype evolution and evolutionary analysis of OsDPE2 locus. Fig. S11: Starch assay and bound the recombinant proteins to OsDPE2 haplotypes. Fig. S12: The binding of recombinant proteins to the maltose assay of OsDPE2 haplotypes using differential scanning fluorimetry (DSF). Fig. S13: Standard curve of endogenous OsDPE2 and transgene OsDPE2(AQ)ZH11 genes. [file 12284_2023_618_MOESM5_ESM.pdf]

Supplemental data

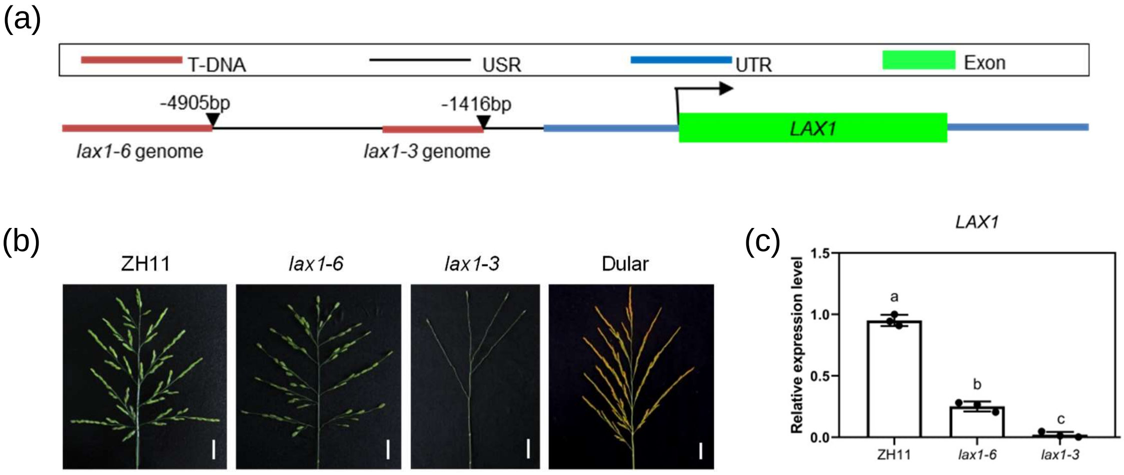

Fig. S1 T-DNA mutants of *LAX1* in ZH11. (a) Diagram of the T-DNA insertion site of *lax1-6* and *lax1-3*. (b) Panicle phenotypic characteristics of ZH11, *lax1-6*, *lax1-3* and Dular. Scale bar of panicle =4 cm. (c) *LAX1* gene expression in 2 mm young panicles of WT, *lax1-6* and *lax1-3*. Ten young panicles were mixed into a biological replicate, and each data group had three biological replicates. Bars represent mean ± SE (one-way ANOVA).

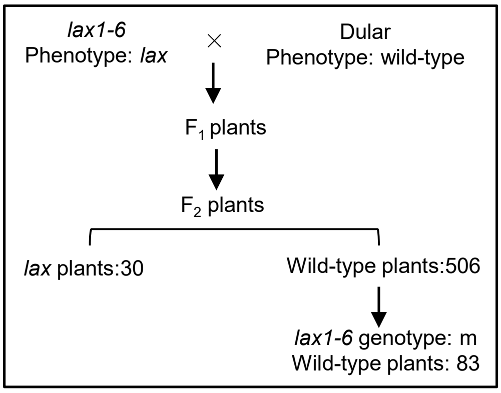

Fig. S2 Panicle phenotype of mutant plants after identifying their *lax1-6* locus. Thirty plants with *the lax* phenotype and 83 plants with like wild-type phenotype were selected for further analysis.

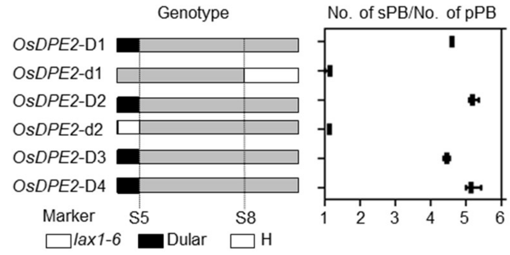

Fig. S3 Genotypic identification and phenotypic observation of the recombinants identified between S5 and S8 via the progeny test.

(a)

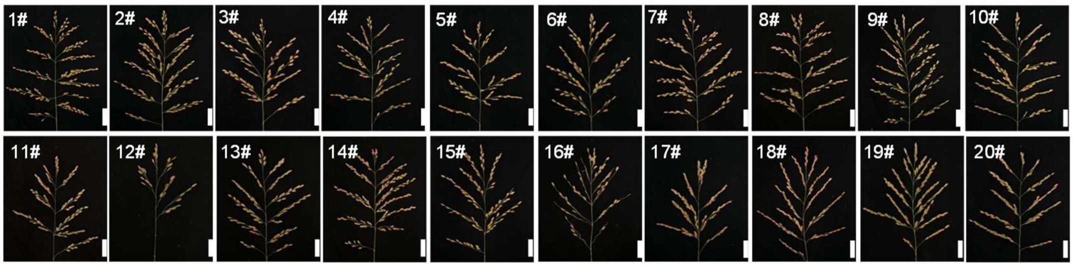

(b)

Genotype \ *OsDPE2*-Com^{lax1-6}	1#	2#	3#	4#	5#	6#	7#	8#	9#	10#
lax1-6	m	m	m	m	m	m	m	m	m	m
OsDPE2-Com	+	+	+	+	+	+	+	+	+	+
Genotype \ *OsDPE2*-Com^{lax1-6}	11#	12#	13#	14#	15#	16#	17#	18#	19#	20#
lax1-6	m	m	m	m	m	m	m	m	m	m
OsDPE2-Com	+	-	+	+	+	-	+	+	+	+

(c)

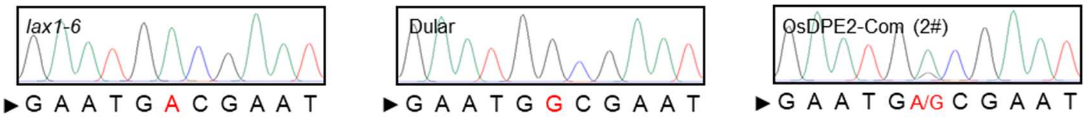

Fig. S4 Phenotypic analysis and positive transgenic detection of the progenies of the *OsDPE2*-Com T_1 in *lax1-6* background. (a) Panicle phenotypic characteristics of progenies of the *OsDPE2*-Com T_1. Scale bar=4 cm. (b) Positive transgenic detection of progenies of the *OsDPE2*-Com T_1 in *lax1-6* background. (c) Comparative sequencing analysis of sequence (*OsDPE2* CDS -2nd ~ 9th) among *lax1-6*, Dular and *OsDPE2*-Com.

(a)

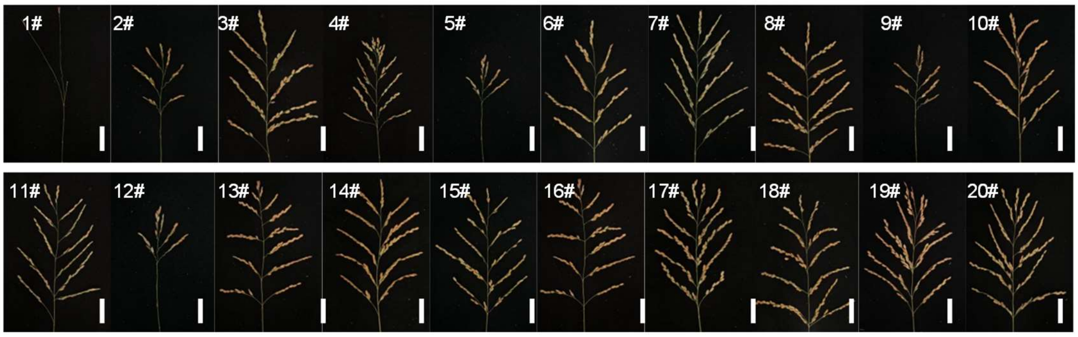

(b)

Genotype \ *OsDPE2*-Com$^{lax1\text{-}3}$	1#	2#	3#	4#	5#	6#	7#	8#	9#	10#
lax1-3	m	m	w	m	m	h	h	h	m	h
OsDPE2-Com	-	+	-	+	+	-	+	+	+	+
Genotype \ *OsDPE2*-Com$^{lax1\text{-}3}$	11#	12#	13#	14#	15#	16#	17#	18#	19#	20#
lax1-3	w	m	h	h	h	w	h	w	h	w
OsDPE2-Com	-	+	-	+	-	-	-	+	+	+

Fig. S5 Phenotypic analysis and positive transgenic detection of the progenies of the *OsDPE2*-Com T_1 in *lax1-3* background. (a) Panicle phenotypic characteristics of progenies of the *OsDPE2*-Com T_1. Scale bar=4 cm. (b) Positive transgenic detection of progenies of the *OsDPE2*-Com T_1. in *lax1-3* background.

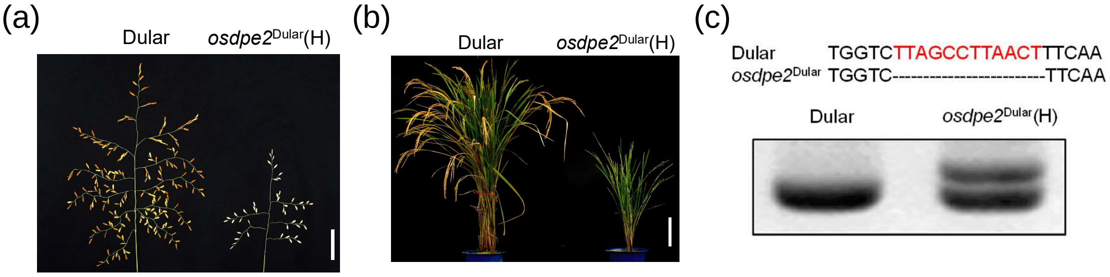

Figure S6 Panicle and plant phenotypic characteristics of Dular and *osdpe2*^{Dular}(H). Panicle (a) and plant (b) phenotypic characteristics of Dular and *osdpe2*^{Dular}(H). Scale bar of panicle =4 cm and plant =20 cm. (c) Identification of *OsDPE2* genotype of WT and *osdpe2*^{Dular}(H).

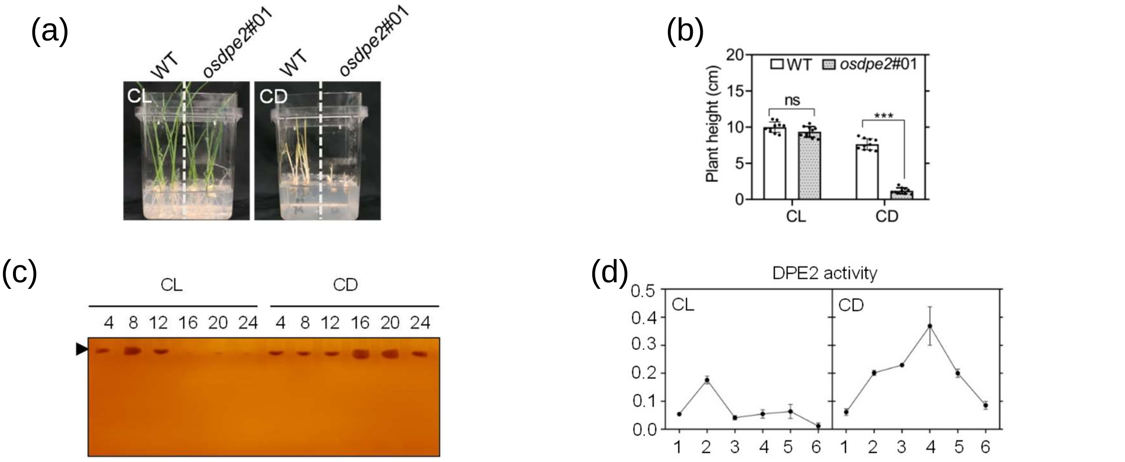

Fig. S7 Growth rate and DPE2 enzyme activity of the plants under continuous light (CL) and continuous dark (CD) conditions. (a) Images of wild-type and *osdpe2*#01 seedlings under CL and CD conditions. (b) Plant height of wild-type and *osdpe2*#01 seedlings under CL and CD conditions. Data are expressed as mean ± SD (n=10 biologically independent samples). Bars represent mean ± standard deviation (one-way ANOVA). (c) In-gel DPE2 enzyme assay and (d) OD values of WT under CL and CD conditions. The arrow indicates the DPE2 enzyme. Three plant leaf samples were mixed into a biological replicate at each time point, and each data group contained three biological replicates. Bars represent mean ± SE.

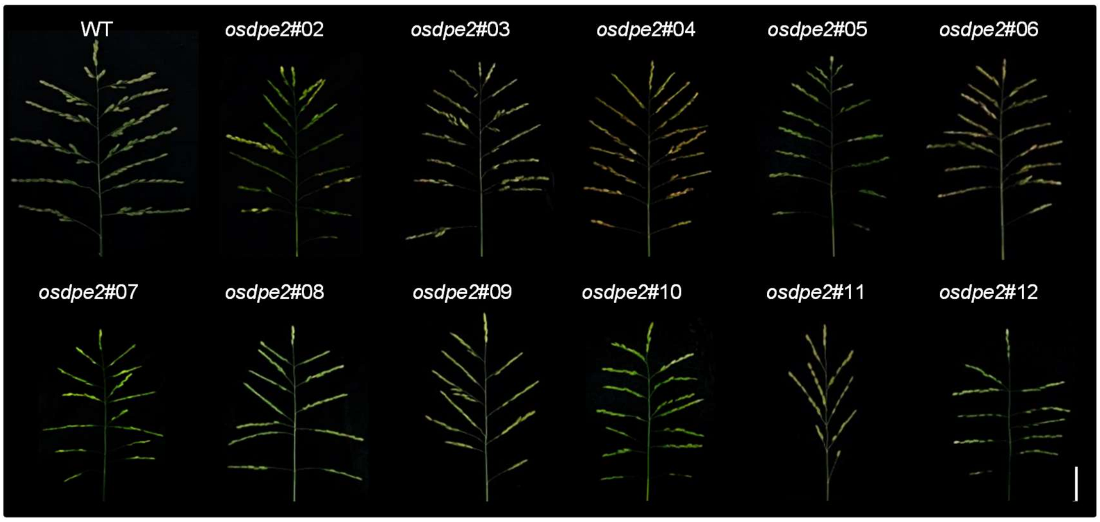

Fig. S8 Panicle phenotypic characteristics analysis of the 12 allelic *OsDPE2* mutants.

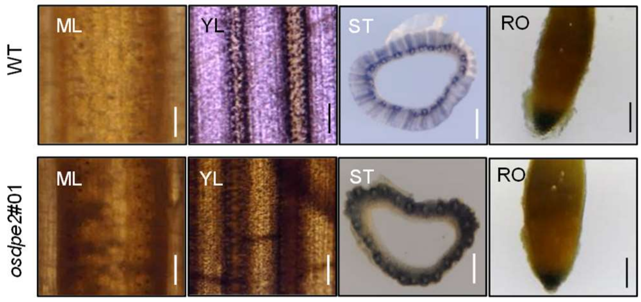

Fig. S9 Iodine staining analysis of various vegetative organs of wild-type and *osdpe2*#01. ML, mature leaf; YL, young leaf; ST, stem; RO, root. Scale bar of panicle =1 mm.

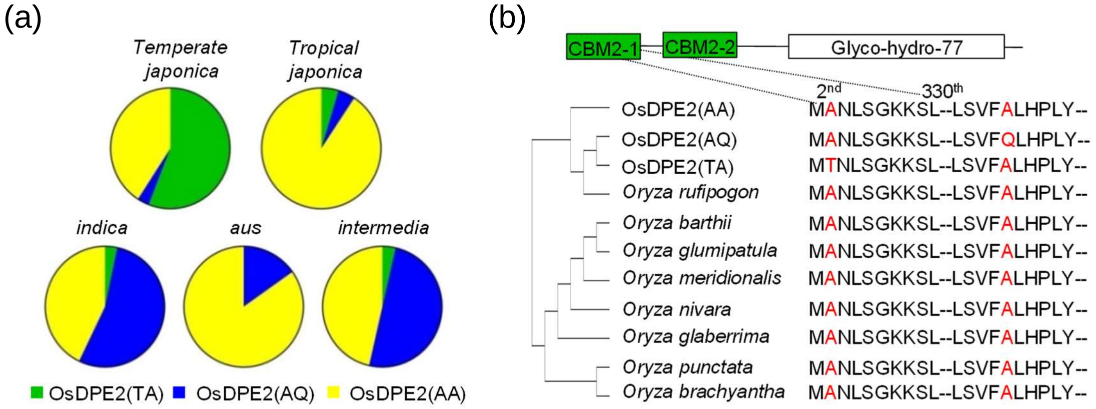

Fig. S10 Haplotype evolution and evolutionary analysis of *OsDPE2* locus. (a) Haplotype evolution analysis of *OsDPE2* locus in cultivated rice (504 samples) from Ricevarmap databases. (b) Phylogenetic analyses of *OsDPE2* in the OsDPE2 haplotypes, wild rice, and other rice germplasm resources using EggNOG v5.0 software.

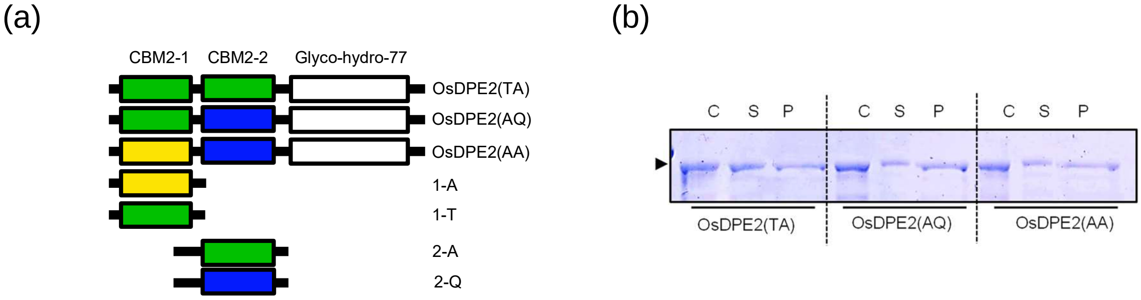

Fig. S11 Starch assay and the recombinant proteins bound to OsDPE2 haplotypes. (a) Diagram of OsDPE2 proteins with different haplotypes of GST tag and proteins with different CBM2 domain of MBP tag. Starch assay and OsDPE2 proteins bound to different haplotypes of GST tag.

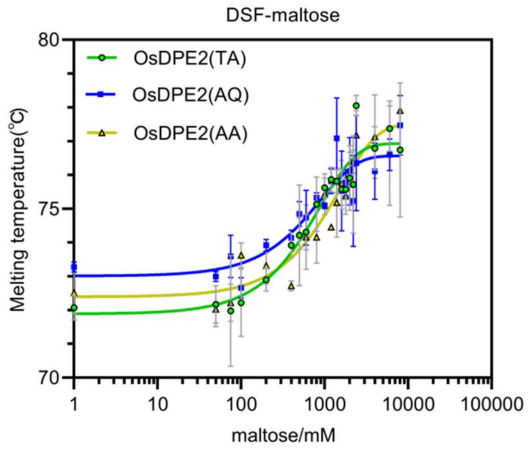

Fig. S12 The binding of recombinant proteins to OsDPE2 haplotypes using differential scanning fluorimetry (DSF). The melting temperature (℃) of each OsDPE2-GST haplotype increased logarithmically with the maltose concentration from 100 to 1000 mM.

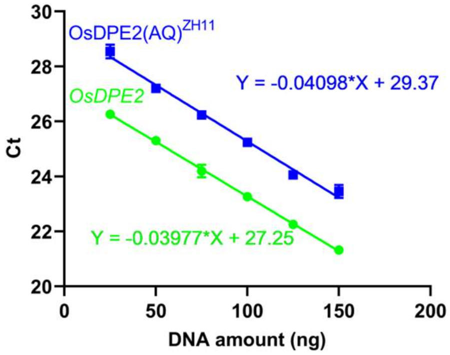

Fig. S13 Standard curve of endogenous *OsDPE2* and transgene OsDPE2(AQ)^{ZH11} genes. Correlation coefficient and slope values were determined. The calculated Ct values were plotted against the total DNA of each starting quantity. Each sample had four replicates.
